# Strengthening India's pandemic preparedness with the four zonal institutes of virology: stakeholder consultations for outbreak response and collaborative research priorities

**DOI:** 10.1186/s40249-026-01484-z

**Published:** 2026-07-28

**Authors:** Kunal Pise, Babasaheb Tandale, Naveen Kumar, Pradip Barde, Ashok Munivenkatappa, Basavaraj Mathapati, Abhijeet Jadhav, Pankaj Rawat, Shrinivasa Basavaraj, Satish Gaikwad, Avinash Deoshatwar, Sakib Akther Pattassery, Susha Kutteyil, Anisha Pulinchani

**Affiliations:** 1 ICMR National Institute of Virology (NIV), Microbial Containment Complex (MCC), 130/1, Sus Road, Pashan, Pune, Maharashtra 411021 India; 2 ICMR National Institute of Translational Virology and AIDS Research, Pune, India; 3 Academy of Scientific and Innovation Research (AcSIR), Ghaziabad, India; 4 National Institute of One Health (NIOH), Nagpur, Maharashtra India

**Keywords:** Collaborative research, Disease prioritisation, Pandemic preparedness, Viral diseases, Stakeholder engagement

## Abstract

**Background:**

The Pradhan Mantri Ayushman Bharat Health Infrastructure Mission (PM-ABHIM) scheme, supported by the World Bank, is establishing four zonal National Institutes of Virology (NIVs) in India. It is essential to identify the zonal disease and research priorities so as to focus research at zonal levels. This study reports a zonal viral disease prioritisation exercise to support outbreak response and collaborative research. It describes zonal-prioritised viral diseases, assesses concordance and differences in zonal priority lists and, and identifies cross‑cutting research themes and preparedness gaps using an multicriteria decision analysis (MCDA)‑based consensus process.

**Methods:**

We conducted a cross-sectional, multi-stakeholder consultative study across four geographical zones of India in March 2024. The zonal-level consultative workshops employed a systematic consensus-building approach using the modified One Health Zoonotic Disease Prioritisation (OHZDP) tool, adapted for emerging viral diseases and applied via MCDA. We identified zonal-level stakeholders, listed 40 viral diseases and categorised them into three groups: common occurrence (Group A), limited occurrence (Group B), and rare occurrences with a chance of emergence, evolution or importation (Group C). The stakeholders from state health departments, medical colleges, research institutes and the Virus Research and Diagnostic Laboratory (VRDL) network ranked the viral diseases and research themes in each zone. Spearman rank correlation was used to assess concordance of priority rankings between zones.

**Results:**

A total of 186 participants (42–48 per zone) contributed to the four zonal workshops including collaborators from VRDL laboratories (26%), public health stakeholders from Integrated Disease Surveillance programme(IDSP) (28%), invited experts (21%), organisers (19%) and Indian Council of Medical Research (ICMR) representatives (6%). The key outcomes included the zone-specific lists of priority viral diseases in Groups A, B and C. There were zonal variations in viral disease prioritisation, reflecting local epidemiological patterns. Spearman rank correlation analysis showed moderate positive correlation between the Central and East zones (rho = 0.697, *P* = 0.031), whereas other pairwise comparisons were not statistically significant, which indicated both shared and distinct priority patterns within the zones. The top five priority viral diseases across zones included dengue, influenza, measles, Japanese encephalitis and hepatitis A. The research themes and subthemes were then decided for collaborative research.

**Conclusions:**

These multistakeholder consultations provided a novel, replicable template for prioritising viral diseases to develop strategies for mitigating the impact of future outbreaks through collaborative research. These zonal priorities may guide similar exercises at national, regional and global levels in future.

**Supplementary Information:**

The online version contains supplementary material available at 10.1186/s40249-026-01484-z.

## Background

Outbreaks of emerging viral diseases have been happening in various zones of India, including severe disease epidemics of Japanese encephalitis in Uttar Pradesh [1, 2], Chandipura virus in Gujarat and Maharashtra [3], Nipah virus in Kerala [4, 5], and the Zika virus in Rajasthan [6, 7]. The COVID-19 pandemic has driven a global focus on virus research. During the COVID-19 pandemic, there were significant regional disparities in disease incidence, the response to the crisis and the distribution of research efforts [8].

The Indian Council of Medical Research National Institute of Virology (ICMR-NIV) in Pune has played a critical role in the nation's war against COVID-19 and has contributed on multiple fronts. The COVID-19 pandemic posed significant difficulties in dealing with such large-scale public health emergencies. There is an ongoing need to improve public health infrastructure and provide required platforms for research on emerging and re-emerging infections/pathogens. It has become important to decentralise the workload at ICMR-NIV by establishing state-of-the-art NIVs at the regional level across the nation.

The Pradhan Mantri Ayushman Bharat Health Infrastructure Mission (PM-ABHIM) plan was announced on October 25, 2021 [9]. The scheme is aimed at strengthening health systems and institutions at all levels of care. The Department of Health Research (DHR), through the ICMR, is creating four Zonal National Institute of Virology units as part of the PM-ABHIM programme to strengthen India's health infrastructure and help manage future pandemics and epidemics.

As per the World Health Organization (WHO) Research and Development (R&D) Blueprint and the Global Health Security Agenda (GHSA) initiatives, disease prioritization frameworks are essential tools for optimizing resource allocation for pandemic preparedness [10–12]. While the WHO uses Delphi techniques and multicriteria decision analysis to identify diseases requiring urgent research and development [13], the One Health Zoonotic Disease Prioritization (OHZDP) tool provides a framework for systematic stakeholder involvement [14, 15].

Under the World Bank's Disbursement Linked Indicator (DLI) 8, ICMR-NIV was mandated to identify and prioritise viral diseases at zonal level, enhance the outbreak investigation capacity of collaborating institutes at zonal level and establish research collaborations. Under this, effective pandemic preparedness required a locally relevant priorities as per the existing epidemiological patterns, healthcare infrastructure and research capabilities. The study aimed to apply a systematic, multicriteria decision analysis (MCDA) based consensus building process to identify zonal-level stakeholders, generate priority list of viral diseases and research themes by collating recommendations at the zonal level, and to describe how this approach can contribute to strengthening pandemic preparedness in India and similar settings.

The aims of the study were to describe zonal prioritised viral diseases, assess concordance and differences in zonal priority lists and, identify cross‑cutting research themes and preparedness gaps using an MCDA‑based consensus process.

## Methods

### Study design and setting

This was a cross-sectional, multi-stakeholder consultative study conducted across four geographical zones of India (North, South, East and Central). It included consultative workshops, organised to bring together multiple experts and potential collaborators from each zone. ICMR-NIV, Pune wanted to identify and prioritise diseases and specific research objectives systematically, by engaging state health authorities, medical colleges, and research institutions in the respective zones.

Workshops were organised for the four zonal NIVs being established at Bengaluru (South zone), Jammu (North Zone), Dibrugarh (East Zone) and Jabalpur (Central Zone). This was conducted under the PM-ABHIM Scheme supported by the DHR through its implementation plan, operational manual and the disbursement linked indicators (DLIs).

**Fig. 1:**
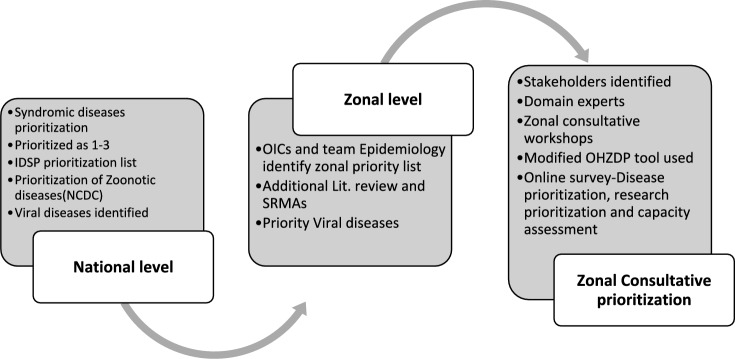
Process adopted for the Zonal Workshop for Viral diseases & Research prioritization, and outbreak capacity assessment for the workshops. *IDSP* Integrated Disease Surveillance Programme, *OICs* Officers in-charge, *OHZDP* One Health Zoonotic Disease Prioritization

The process flow for the organisation of these workshops is showcased in Fig. 1.

### Participants

Participants were purposively selected to represent key institutional stakeholders for viral disease surveillance, research and outbreak response in each zone. The scientists from the Department of Epidemiology and the Officer in-charge of Zonal NIVs started preparation five months before the scheduled workshops. The major task was to identify stakeholders, collaborators and policymakers in these states from each zone. Diverse perspectives were intentionally considered prior to partner identification to promote inclusivity in the activity. Institutions identified included regional-level medical colleges (government and private), central institutes, institutes of national importance, Indian Institutes of Technology (IITs), All India Institutes of Medical Sciences (AIIMS), and veterinary institutes and laboratories within the Virus Research and Diagnostic Laboratory (VRDL) network. For each state, two institutes were shortlisted based on clinical departmental infrastructure, outbreak investigation capacity and laboratory technical strengths, and invited to participate in coordination with their respective state health department.

**Fig. 2:**
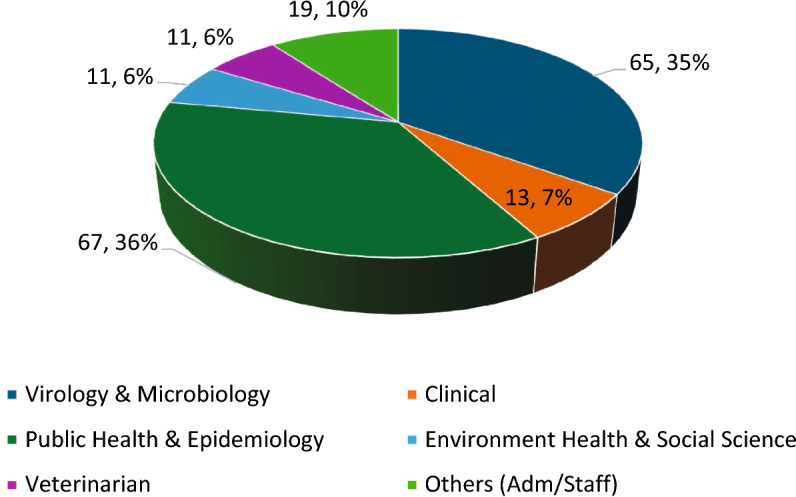
Distribution of participants according to expertise for the zonal viral disease research prioritization workshops [number, percentage is reported]

Additionally, domain experts form clinical medicine, epidemiology and public health, microbiology, environmental health and veterinary science, along with representatives from national and international organisations (e.g. NCDC, WHO), were invited to contribute technical inputs and facilitate the discussions (Table 1). In total, 186 participants (42–48 per zone) attended the workshops, including collaborators from the VRDL laboratories (26%), public health stakeholders [16] from Integrated Disease Surveillance Programme(IDSP) (28%), the invited experts (21%), organisers (19%) and Indian Council of Medical Research (ICMR) representatives (6%) (Fig. 2).

**Table 1:** Role of the participants in the four zonal workshops for viral disease prioritisation, India, March 2024

Type	Central	East	North	South	Total
Collaborators	13 (27%)	12 (25%)	11 (26%)	13 (27%)	49 (26%)
Invited experts	11 (23%)	8 (17%)	10 (24%)	10 (21%)	39 (21%)
ICMR headquarters & nominated experts	6 (13%)	2 (4%)	3 (7%)	0 (0%)	11 (6%)
Organizers	7 (15%)	8 (17%)	7 (17%)	13 (27%)	35 (19%)
Stakeholders	11 (23%)	18 (38%)	11 (26%)	12 (25%)	52 (28%)
Total	48	48	42	48	186

All prior logistical arrangement were coordinated and managed by ICMR-NIV, Pune. Separate ethical approval for this study was submitted to and granted by the Institutional Ethical Committee (IEC).

### Prioritisation methods and processes

#### Phase I

##### Developing list of viral diseases

The core team at ICMR-NIV had prepared an initial comprehensive list of viral diseases based on the ICMR-DHR list, the Integrated Disease Surveillance Project (IDSP) list, literature review, and discussions with different stakeholders, regional units of NIV, and various virus research and diagnostic laboratories (VRDLs). In addition, the team incorporated the national-level of high-priority disease syndromes and associated pathogens provided by ICMR headquarters. Both lists were thoroughly reviewed and a list was prepared for presentation at the workshop. In this prioritization exercise, the nodal officers of the four zonal ICMR-NIVs worked with epidemiology and public health scientists at ICMR-NIV to draw up a comprehensive list of viral diseases. Forty viruses were listed, including those associated with outbreaks commonly reported in India (*n* = 10), others with limited outbreak potential (*n* = 15) and the remaining with the potential to cause outbreaks due to risks of emergence, evolution, and importation (*n* = 15). The list of viral diseases for the prioritization exercise is mentioned in Supplement 2. It consisted of three categories: *Group A (Common Occurrence); Group B (Limited Occurrence) and, Group C (Chance of Importation).*

The diseases categorization was based on the epidemiological review of India-specific surveillance data and outbreaks, so as to ensure focus on regional, rather than global patterns.

##### MCDA criteria and scoring tool

The epidemiology team at NIV developed an MCDA-based scoring tool adopted from the established OHZDP and WHO disease prioritization methodologies [13, 17]. The weighted questions were designed to evaluate candidate viral diseases against five predefined core criteria. These criteria were adopted from a similar OHZDP activity conducted by the National Center for Disease control (NCDC), India in 2020 [17]. All criteria-related assessment questions were adopted to the local zonal epidemiological context, and distributed to all participants for pre-workshop response collection using Microsoft Office forms.

The criteria for disease prioritization include: (1) severity of disease in humans (22% weight), (2) capacity for prevention and control (15% weight), (3) potential for introduction or increased transmission in the region (20% weight), (4) socio-economic impact (18% weight), and (5) pandemic/epidemic or outbreak potential (25% weight). These weights were determined based on findings from a comparable 2020 zoonotic diseases prioritization workshop [18] and prior consultation within research team including public health specialists, NIV epidemiologists and virologists, and applied uniformly across zones.

##### Pre-workshop data collection

Data from public health agencies and a scoping review of literature on platforms like PubMed and Google Scholar provided insights into prevalent viral diseases. Information from the WHO, CDC, NCDC, and IDSP was also reviewed. This background information helped facilitate workshop discussions for research prioritization. Information on each state’s capacity to respond to outbreaks or epidemics was collected prior to the workshop, compiled, and presented during the workshop.

The diseases were prioritized by the potential participants of the workshops individually as a survey questionnaire, which include the policymakers, subject experts, public health specialists, clinicians and virologists. A pre-workshop tool for research prioritization was circulated amongst participants in the workshop to understand the primary thematic areas of research for the identified priority viral diseases in the respective zone.

#### Phase II

Using Microsoft Office Forms, a questionnaire based on the developed criteria were deployed to collect online responses from the prospective workshop participants individually, forming the foundation for consensus-building during the workshop. The questionnaire comprised 25 structured questions across five criteria domains, which enabled pre-workshop analysis of expert opinions and identification of areas requiring focused discussion during consensus-building sessions. Each participants received the questionnaires with an explanation of the purpose of the survey, expectations from them and the methodology of scoring the said questions. All responses were compiled by the team in advance and presented at the time of the workshop to generate consensus about priority viral diseases for the states and then for the zones.

#### Phase III

##### Implementation and consensus building

During the workshops, a structured prioritization tool was used to identify priority viral diseases and corresponding research areas through a collaborative, team-based exercise with expert inputs. It was expected that through the structured questionnaire/tool, priority viral diseases could be identified by the participants of the survey. Viral diseases were ranked for Group A, B and C. ‘A’ were those ones which were of common occurrence and public health importance, ‘B’ were those significant and having limited occurrence and ‘C’ included that having a chance of importation into the state/country. All the state team were provided with the observation of the online surveys, the relevant occurrence of outbreaks in the states, the relevant information and online sources for these diseases. Priority was given to the first five identified viral diseases in the zonal workshop to identify the research areas for them.

The workshops were conducted in March 2024 (Supplement 3). Each workshop followed a structured two-day agenda: Day 1 focused on disease prioritization using modified Delphi techniques, with presentation of pre-workshop survey results, state-wise ranking exercises and facilitated consensus-building. Experts from various field helped state teams to work upon the prioritization of viral diseases, followed by finalisation at zonal level.

Day 2 emphasized research priority identification through expert working groups, organized by thematic areas (virology, clinical, epidemiological and environmental).

**Fig. 3:**
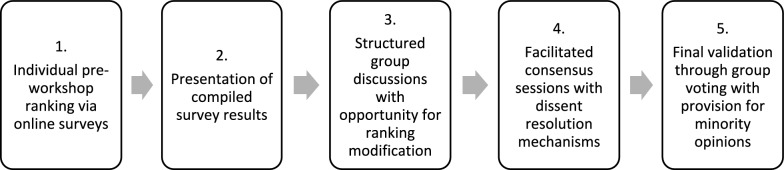
Steps in the consensus building process

We used a hybrid approach, in which we combined Delphi methodology with face-to-face deliberation [19, 20]. All participants were encouraged to participate, regardless of seniority or institutional affiliation (Fig. 3). In each zone, prior ranking from pre-workshop survey were presented, followed by state-wise small group discussions to review the epidemiological evidence and context of each disease. The facilitators invited justification from different states, to clear any discrepancies in rankings. Consensus was achieved through open discussion. If there still were any disagreement for two rounds of discussions, a majority vote was adopted by the zonal group.

### Statistical analysis

Disease-level outputs (e.g., prioritization scores or rankings) were summarized descriptively to reflect stakeholder inputs [experts, interinstitutional non-governmental organizations (NGOs)-WHO, IDSP] across consultations and were not subjected to hypothesis testing.

Inferential analysis was limited to assess the concordance between geographic zones. Specifically, pairwise associations between zone level rankings were evaluated using Spearman's rank correlation coefficient. Statistical significance for these correlations was tested using standard procedures for Spearman’s rho, with a predefined significance threshold (*P* < 0.05).

This approach was chosen because the primary objective was to examine consistency in prioritization patterns across zones rather than to test differences between individual diseases. Participant characteristics and workshop feedback were summarised as counts and percentages for categorical variables. Feedback scores collected on 0–5 Likert‑type scales are presented as median and Inter-quartile range (IQR). All analyses were performed using R version 4.5.1 (R Foundation for Statistical Computing, Vienna, Austria).

## Results

### Prioritised lists of viral diseases

The participants of the various zone were helped to prioritise the virus disease of concern in their respective zones through a consultative process. Results of the online survey were shared with them and they were asked to rank the viral diseases in their states. Each state presented their prioritised list and consensus was generated for the zonal priority list of viral diseases of importance. Table 2 represents the list of the zonal level priority viral diseases.

**Table 2:** Zonal priority list of Group A, B and C viral diseases in four regions of India

Rank	Central zone	East zone	South zone	North zone
	A (Common occurrence)			
1	Dengue	Dengue	Dengue	Influenza*
2	Measles	Japanese encephalitis*	Influenza	Dengue
3	Japanese encephalitis	Influenza	Measles	Hepatitis A
4	Influenza	Measles	Japanese encephalitis	Measles
5	Hepatitis A	COVID-19	Hepatitis A	Rotavirus*
	B (Limited occurrence)			
1	Chickenpox	Chickenpox	KFD	Chickenpox
2	Mumps	Rabies	Zika	Mumps
3	Hepatitis C	Hepatitis C	Nipah	Respiratory syncytial virus (RSV)
	C (Chance of importation)			
1	Zoonotic influenza	Zoonotic influenza	Rabies	Zoonotic influenza
2	Herpes simplex	Novel coronavirus	West Nile	Novel coronavirus
3	Novel Coronavirus	Herpes Simplex	Polio	

^*^in Table 2 denote descriptive regional differences in rank (pre-specified as a difference of ≥ 4 rank positions between any two zones) and should not be interpreted as results of inferential statistical tests

Statistical analysis revealed regional variations in disease prioritization. Spearman rank correlation analysis showed that there is positive correlation between East and Central Zones (rho = 0.697, *P* = 0.031) while correlations between other zone pairs were weaker and not statistically significant. We calculated Spearman rank correlation coefficients for each pair of zones for the ranked Group A lists for zonal comparison of overall priority lists. Given that the number of diseases per group and per zone was small and rankings were derived through expert consensus rather than independent measurements, we did not perform formal hypothesis testing at the level of individual diseases.

Interestingly some regional patterns emerged: Japanese encephalitis ranked significantly higher in East zone (2nd position) compared to North zone (8th position), reflecting higher endemicity in northeastern states. While Influenza was prioritised in North zone (1st position), the Central zone had ranked it at 4th position, which reflects different seasonal patterns and healthcare infrastructure priorities in these zones.

### Cross cutting research priorities

For each zone, working groups identified thematic and sub thematic research areas for the first five Group A diseases. Table 3 depicts the five cross-cutting themes applicable across the four zones: (1) diagnostics and surveillance (including genomic and syndromic surveillance); (2) vaccine development, deployment and effectiveness studies; (3) vector, reservoir and environmental studies; (4) clinical management and therapeutics; and (5) health systems and policy research, including outbreak preparedness and response. The details of the zone wise thematic and sub-thematic research areas prioritised, such as Japanese encephalitis-focused research in the East zone and influenza-related research in the North zone are represented in the supplemental file no.5.

**Table 3:** Cross-cutting research themes for priority viral diseases across four zones

Research theme	Priority areas	Zones emphasizing
Diagnostics & surveillance	POC tests, genomic sequencing, early warning systems, digital surveillance	All zones
Vaccine development	Universal vaccines, delivery mechanisms, coverage studies, effectiveness evaluation	All zones
Vector-environmental	Climate change impacts, vector control, One Health approaches, environmental monitoring	Central, South, East
Clinical therapeutics	Antivirals, severity biomarkers, treatment protocols, clinical management	All zones
Health systems research	Implementation science, health economics, community engagement, policy analysis	All zones

### Outbreak investigation support gaps and capacity building for outbreak investigation

The participant described the existing outbreak-response capacities within their respective states and identified common challenges, which was organized into five thematic areas represented in Table 4: (i) the need of early warning systems, (ii) community information and resilience, (iii) the need for Research and Development, (iv) Public health infrastructure and Human Resource, and (v) Others.

i. Need for early warning system: the group shared that there were challenges in ensuring surveillance through the IDSP-IHIP (Integrated disease surveillance Programme-Integrated Health Information Platform) reporting system at the district surveillance units (DSU) in most of the states, as it was relatively new. To emphasize this, the importance of a real-time village-level reporting system was highlighted. Also, it was shared that regularity in the DSU meetings, timely acknowledgment of outbreaks and establishment of emergency operations would be needed to improve the reporting. Bringing in the concept of One Health, some of the experts shared views regarding improved joint reporting in the National Programme on Climate Change and Human Health (NPCCHH) to understand the need to identify potential outbreaks lurking in the animal and human worlds. Similarly, models for human, animal and environmental interactions and the effects of climate change would be a necessary part of the surveillance network. However, it was felt that it would be a challenge to identify the sub-clinical cases at the village level. It was suggested that community participation be integrated in the IHIP, and research and development on this aspect was indicated. For the state that had international borders, it was suggested that the regular traveller screening to be integral part of the surveillance. It was common sentiment that the existing surveillance system needs strengthening than introducing new ones [21].
ii. Community information and resilience: community engagement in surveillance was echoed by the participants as need of the hour, since only than a real time reporting was said to be practical and of use. Also, addressing misinformation in public was important not only to protect public health emergency preparedness but also to improve community resilience [22, 23]. It was suggested that health care workers and community volunteers would play a pivotal role in sensitizing the community about outbreak preparedness and surveillance. This would be especially required to clear the misinformation and disbeliefs in the community regarding prevention and control measures targeting pathogens and vectors, such as vaccination. The effective use of social media was highlighted as a useful medium for communication in such situation and optimal utilization of this resource would add value to the surveillance and outbreak response, this was similar to the observations of Hadi, Tamer in 2014 [24].
iii. Public health infrastructure and Human Resource: many of the states observed that there is a need for the well-equipped public health laboratories with trained human resources to improve the diagnostic arm of outbreak response. Along with the laboratory, discussion around ensuring the quality assurance mechanisms was re-iterated by many of the participants of the zones. Some of the states faced vacancies for the full-time epidemiologist, DSO/DEO/microbiologist in their state/district surveillance units. Similarly, emphasis was laid upon the infection control practices in the hospital and triaging for clinical management and need for improvement. Localised public health human resource capacity building was suggested in the discussion like other studies [23].
iv. Research and development: it was observed that a common felt need across all the zones was to have support regarding research and development from the institutions like the ICMR-NIV. Various important areas that were suggested during the discussion of the challenges were- institutional level capacity building for R&D; whole genome sequencing for virus disease outbreaks; funding and grant access for projects of common interest in virus research; further research in emergence of new viruses; effective use of mobile technology and patient tracking and systematic surveillance of data available on wildlife, pets and vectors in the four zones under the proposed ICMR-NIVs.
v. Others: some of the important other challenges especially for those states with international borders, were traveller screening as a part of surveillance. The need for utilisation of artificial intelligence in surveillance of virus disease of concern in the zone could be helpful in the near future [25]. A One Health approach to enhance surveillance would be needed, e.g. waste water surveillance [26]. Incentivisation for services, especially for community volunteers, was suggested. During the outbreak and as a part of response, it was added that psychological help in form of bereavement counselling and guidance [27] through NGOs working in that area may add value.

**Table 4:** Common zonal challenges for surveillance and epidemic/pandemic preparedness

Zone	Common challenges				
	Early warning systems	Community information and resilience	Research & development	Public health infrastructure & HR	Others
East	• Delay in outbreak response due to accessibility of data • Acknowledgement of Event alerts for timely action	• Community engagement & awareness on outbreak roles • Real time reporting at village level	• Virus genome sequencing • Involvement of medical colleges	• Well- equipped labs with trained staff • Training of health-care workers	• Preparing in advance for outbreaks • Preparedness for common outbreaks
Central	• Reporting from private sector • Identification of subclinical and mild clinical cases	• Community engagement • Real time reporting at village level	• Genome sequencing • Involvement of medical colleges	• Well- equipped labs with trained staff • No. of labs need to be increased	• Preparing for future outbreaks • Emergence of novel viruses
North	• Reporting from community members • Traveller’s screening	• Community engagement • Community reporting	• Multisectoral collaboration • Patient roles for tracking and control	• Health care worker sensitisation • Private practitioner involvement	• Incentives for community health worker/volunteers to report • Lack of clinical data
South	• Active surveillance of human cases, vectors, other hosts and the environment • Lacking digital data entry system	• Community involvement in surveillance and reporting • Lack of technical tools	• Disease modelling system • Systematic surveillance and data on wildlife, pets and vectors	• Preparedness on laboratory staff training needs • Training of health and laboratories staff	• Support from non-governmental organizations • Environmental aspects—animal hosts, wildlife and livestock investigations with humans

The participants provided their workshop evaluation and feedback and suggestion to the team as mentioned in supplement 6 and 7.

## Discussion

This study adds to the disease prioritization methodology by demonstrating the systematic application of multicriteria decision analysis at the sub-national level. In our approach we tried to address limitations that were identified in previous prioritization exercises by: (1) incorporating regional epidemiological variations, (2) ensuring balanced stakeholder representation, (3) providing a transparent and reproducible methodology and (4) integrating research priority identification with disease prioritization. This hybrid methodology establishes a balanced approach between pure survey-based or pure discussion-based prioritization exercise. This helps capture expert knowledge and collective wisdom.

### Similarities with international frameworks

Through our experience, we found that the output aligns with the global health security priorities. The viral diseases prioritised matched with those of the WHO R&D Blueprint diseases, particularly for Group C diseases (Crimean-Congo haemorrhagic fever, Nipah virus, Rift Valley fever) [10, 11]. Even the capacity building priorities identified in the workshops, closely resemble that of GHSA Action Packages, especially surveillance strengthening, laboratory systems enhancement, and workforce development [11, 12, 28]. We feel that a multizonal approach is more suitable as it offers unique advantages for large, diverse countries compared to the single-area prioritization method. It might prove to be a useful method for diverse countries like India.

### Need of regional epidemiological insights

Variations in zonal virus priorities reflect real epidemiological differences between these zones. For example, Japanese encephalitis prioritization in the East zone, is indicative of the higher incidence rates in the northeastern states of India, while influenza, has been prioritized in North Zone because of the observed seasonal pattern of the disease in this region. As opposed to uniform national strategies, zonal approaches address regional disease patterns and healthcare infrastructure variations that are more suitable for pandemic preparedness.

### Implications for future research and practice

The collaborative workshops conducted at zonal NIV in India hold importance for the future of research and practical application, in managing diseases within each zone. To begin with the identification of areas tailored to the unique epidemiological landscape of each region emphasizes the significance of localized strategies in combating viral diseases [29]. This underscores the need for research on region-specific viral pathogens and risk factors to shape targeted prevention and control measures. Additionally, prioritizing diseases for each zone offers a roadmap for research initiatives guiding allocation of resources and focus areas for further study [30]. The active involvement of stakeholders from health departments, experts and academicians highlights the value of collaboration in addressing public health issues. It stresses the importance of maintaining partnerships between researchers, policymakers, and healthcare professionals to ensure the implementation of research findings into practice. Finally these workshops provide a platform for exchanging knowledge and enhancing capacities promoting readiness and resilience against emerging and recurring diseases [31]. Therefore, future research endeavours should concentrate on strengthening collaborations, conducting region-specific studies, and translating research outcomes into actionable policies and interventions to effectively reduce the impact of viral diseases in each zone.

Due to the identification of cross-cutting research themes (Table 3), a foundation for inter-zonal collaboration is possible, while allowing zone-specific emphasis**.** This approach will help optimize the resource utilization by using the research infrastructure and expertise which are pertinent to zonal-level needs.

### Ensuring implementation and sustainability

The success of this virus disease and research prioritization exercise, will depend upon its sustainability and institutionalisation. As per the detailed action plan for each zonal NIV, we have included the following to ensure sustainability: (1) integration into the zonal NIV mandates with dedicated budget allocations under PM-ABHIM, (2) annual research funding calls aligned with established priorities, (3) formal collaborations with identified partners-through an MoU, (4) quarterly progress monitoring and annual priority updates, through an independent verification agency. Through the inclusion of the government partnerships and the existing ICMR research agenda/programme, we intend to ensure sustainability beyond the project support period. Since then, based on the virus disease prioritization and research theme identification, the local-level collaborators have been helped by ICMR-NIV to develop pertinent research.

There are some limitations to the study. The workshops were designed and facilitated by ICMR-NIV, which also has four new zonal houses coming up. Despite our efforts to engage a wide range of external collaborators, our institutional perspective may have influenced the agenda. However, ICMR-NIV was a catalytic agent for the viral disease prioritization and zone-led research. Only 80 of 186 participants (43%) provided their complete structural feedback, which might bring in response bias. We did not measure the post-workshop changes in surveillance performance, outbreak response, or collaborative research outputs, as they are planned in upcoming phase III and IV of the PM-ABHIM’s DLI-8 initiative.

## Conclusions

Developing upon the regional epidemiological insights and cross-cutting research themes identified in this study, the zonal consultations provided a practical example of how structured MCDA-based prioritisation can be applied at the sub-national level in a large, diverse country. The collaborative workshops conducted at the zonal NIVs represent an important step in laying the groundwork to strengthen India's readiness against viral diseases by identifying zone-specific priorities and garnering collaboration among diverse stakeholders. Through these workshops, unique research areas specific to the needs of the local epidemiological landscape were highlighted to address local viral diseases. By prioritizing viruses and related research initiatives, more suitable resource allocation can be earmarked to ensure more optimal outbreak preparedness and response. The active engagement of researchers, policymakers and healthcare professionals accentuates the value of developing and maintaining the partnerships for effective implementation of findings of the research projects. The workshops provided a common platform for the exchange of the knowledge and helped enhance the capacities of the collaborators. This promoted readiness and resilience against emerging and recurring diseases. International alignment with WHO R&D Blueprint and GHSA frameworks showcases the global relevance of this approach and thus its potential applicability to other countries facing similar challenges. Post-workshop activities planned include developing action plans, MOUs with collaborators, capacity building and collaborative research. The ultimate goal is to translate these outcomes into some implementable strategies to mitigate the impact of future viral outbreaks; however, this study reports the prioritisation process and immediate outputs rather than the future implementation outcomes. More importantly, we showcase the experience in adopting and implementing the OHZDP workshops at a sub-national level for virus disease and research prioritization led by regional researchers, programmers and development partners. It also suggests the possibility of replication in similar geographies elsewhere. We feel that this example is flexible, futuristic and dynamic based on the needs of the zones.

## Supplementary Information

Supplementary Material 1

## Data Availability

No datasets were generated or analysed during the current study.
