# Supplementary material for: Strengthening India's pandemic preparedness with the four zonal institutes of virology: stakeholder consultations for outbreak response and collaborative research priorities
Source: Infect Dis Poverty. 2026 Jul 28;15:84. doi: 10.1186/s40249-026-01484-z (PMC13411115; doi:10.1186/s40249-026-01484-z)
Supplement: Supplementary file 1 — Supplementary Material 1 [file 40249_2026_1484_MOESM1_ESM.docx]

Contents

[**Supplement 1-** Zonal distribution of states 1](#_Toc232712132)

[**Supplement 2-**Draft list of viral diseases for prioritization 1](#_Toc232712133)

[**Supplement 3-** Schedule and participation in the Zonal Workshops for Virus Disease and research prioritization 2](#_Toc232712134)

[**Supplement 4-** Distribution of the Participants in the Zonal Workshops 2](#_Toc232712135)

[**Supplement No. 5-** Zone wise list of themes and subthemes prioritised for research areas. 3](#_Toc232712136)

[**Supplement 6-** Overall comment and Suggestions for improvement 5](#_Toc232712137)

[**Supplement 7-** Workshop Evaluation and Feedback 6](#_Toc232712138)

[**Supplement 8-**List of experts and their affiliations 7](#_Toc232712139)

[**Supplement 9-** Post workshop activities 9](#_Toc232712140)

**Supplementary files-**

## **Supplement 1-** Zonal distribution of states

## **Supplement 2-**Draft list of viral diseases for prioritization

| **A**  **(Common Occurrence)** | **B**  **(Limited Occurrence)** | **C**  **(Chance of Importation)** |
| --- | --- | --- |
| Chikungunya | Adenovirus Associated diseases | Buffalopox/Cowpox/Camel pox |
| COVID 19 | Crimean-Congo haemorrhagic fever (CCHF) | Ebola virus disease/Marburg Virus |
| Dengue | Chandipura virus Encephalitis | Hand foot and mouth disease associated with EV71 |
| Hepatitis A | Chickenpox | Hendra Virus Disease |
| Hepatitis E | Hepatitis B | Hanta Virus Disease |
| Influenza | Hepatitis C | Herpes Simplex |
| Japanese Encephalitis | Hand foot and mouth disease associated with Coxsackie Virus | Lassa fever |
| Measles | Human papilloma virus associated cancers | Middle east respiratory syndrome (MERS) |
| Rotavirus Diarrhoea | Kyasanur Forest Disease (KFD) | Novel Coronavirus |
| Rubella | Monkey Pox | Poliovirus |
|  | Mumps | Rift Valley Fever |
|  | Nipah virus infection | Smallpox |
|  | Rabies | West Nile Virus |
|  | Respiratory syncytial virus (RSV) | Yellow Fever |
|  | Zika | Zoonotic Influenza |

## **Supplement 3-** Schedule and participation in the Zonal Workshops for Virus Disease and research prioritization

| Sr No | Zone | Place | Dates | No. of participants |
| --- | --- | --- | --- | --- |
| 1 | **North** | Jammu | 22nd-23rd March 2024 | 42 |
| 2 | **South** | Bengaluru | 12th-13th March 2024 | 48 |
| 3 | **Central** | Bhopal | 15th-16th March 2024 | 48 |
| 4 | **East** | Guwahati | 19th-20th March 2024 | 48 |
|  |  |  | **TOTAL** | **186** |

## **Supplement 4-** Distribution of the Participants in the Zonal Workshops

| Speciality | Central | East | North | South | Total |
| --- | --- | --- | --- | --- | --- |
| Virology & Microbiology | 19(40%) | 20(42%) | 16(38%) | 10(21%) | 65(35%) |
| Clinical | 4(8%) | 3(6%) | 1(2%) | 5(10%) | 13(7%) |
| Public Health & Epidemiology | 15(31%) | 17(35%) | 18(43%) | 17(35%) | 67(36%) |
| Environment Health & Social Science | 4(8%) | 1(2%) | 0(0%) | 6(13%) | 11(6%) |
| Veterinarian | 3(6%) | 2(4%) | 2(5%) | 4(8%) | 11(6%) |
| Others (Admin/Staff) | 3(6%) | 5(10%) | 5(12%) | 6(13%) | 19(10%) |
| Grand Total | 48 | 48 | 42 | 48 | 186 |

## **Supplement No. 5-** Zone wise list of themes and subthemes prioritised for research areas.

| **South Zone** | | | | | |
| --- | --- | --- | --- | --- | --- |
| **Diseases** | **Virology** | **Clinical** | **Epidemiological** | **Other** | |
| Dengue | Discovery: biomarkers, genetic susceptibility | Development: Antiviral trials | Development: AI-based disease modelling using multi-sectoral data | Development: Virus detection in mosquitoes and improved surveillance tool for early warning system | |
| Influenza | Discovery: Genetic susceptibility, Universal influenza vaccine | Development: Nasal vaccine | Discovery: Potent, stable and cost-effective vaccine | Delivery: Sentinel surveillance in migratory birds, domestic and wild animals in the zone | |
| Measles | Development: Inactivated measles vaccine | Development: Field studies for POC test | Description: Age shift patterns | Discovery: Performance of vaccine-vaccinated and non-vaccinated | |
| Japanese Encephalitis | Discovery: POC/ Diagnostic | Development: Vaccine development | Description: Epizootic pathways, transmission and reservoirs in different context | Delivery: Efficacy of JE vaccine | |
| Hepatitis A | Discovery: New disinfectant for mass use | Development: POC molecular test, oral vaccine | Discovery: Oral vaccine in disease prevention and control | Discovery: Studies on water and migratory birds | |
| **Central Zone** | | | | | |
| **Disease** | **Virology & Immunology** | **Clinical** | **Epidemiological** | **Environmental** | |
| Dengue | Delivery: implementation of algorithm and tools for dengue diagnosis | Development: management protocols | Delivery: Community engagement | Descriptive: Climate variability & vector dynamics | |
| Measles | Delivery: NGS to track transmission of measle cases | Descriptive: Booster dose studies | Delivery: vaccine acceptance and coverage | Nil | |
| Japanese Encephalitis | Descriptive: One Health approach for JE management (birds & Pigs) | Delivery: early access for effective treatment | Delivery: Surveillance improvement | Development: protocol for One Health Surveillance | |
| Influenza | Discovery: universal vaccines | Description: postinfection sequelae | Development: Early warning system | Description: Indoor pollution (AQI) and its association with infection | |
| Hepatitis A | Development: POC test | Descriptive: comorbidities affecting liver | Delivery : Hygiene& Sanitation | Delivery: Hygiene & Sanitation/sewage surveillance | |
| **East Zone** | | | | | |
| **Diseases** | **Virology** | **Clinical** | **Epidemiological** | **Environment** | **Immunology** |
| Dengue | Discovery - Virus host interaction (Immunotherapy) | Development biomarkers of severity, drug targets and immune checkpoint modulators / delivery of a triple layered protocol for early diagnosis, early referral and quality management/ Immunomodulators | Description / Delivery: Identification of risk group for complications | Development: Early warning system (Integrated approach) | Development- Vaccine candidate (All serotypes- Vaccine) |
| Japanese Encephalitis | Description - zoonotic scenario, mode of spread, vectors (if any variation in East/NE-(Zeno-surveillance) | Delivery-prioritization of risk group for vaccination | Description: Distribution of disease (agent, host, vector)-Change in *AGE Group* | Development -Advanced vector control strategies | Development- POC & RDT (Early identification) |
| Influenza | Description - changing strains will provide vaccines easily Indigenous | Delivery-vaccine trials high risk groups/Pan-influenza Vaccine trial | Delivery: Strengthening of surveillance (International Border) | Description- Monitoring of non-human hosts for emergence of new strains / Delivery: Improved methods awareness (One health Approach) | Description-Virus host interaction (Biomarkers for Severity) |
| Measles | Discovery - Breakthrough infections | Discovery- Non-Live Vaccine | Delivery: Vaccine efficacy (Implementation Research) | Nil | Description- Sero-surveillance (Susceptible Adults-Adult Vaccination) |
| COVID-19 | Delivery - Vaccine prevented further pandemics (Efficacy, Sustainability, or newer ones) | Development-Immunotherapy | Description: Identification of new variants (WGS) + NCD Increase in any risk | Discovery: Origin/animal reservoir/ intermediate host (Zoonoses) | Descriptive- Virus host interaction (Biomarkers for Long COVID, MISI-C/A) |
| **North Zone** | | | | | |
| **Disease** | **Virological** | **Clinical** | **Epidemiological** | **Immunology** | |
| Influenza | Description - Molecular Epidemiology-New Variants | Delivery-  Outcome studies  Increasing acceptability of vaccine, Vaccine coverage, Health literacy affordability, Community engagement | Description-  Surveillance  Mapping susceptible pop. | Discovery-  Virus Host Interaction  Vaccine Candidates, universal vaccine | |
| Dengue | Discovery-Vaccines/Antivirals  Novel Antiviral, Human MAB | Description - Disease course and progression Studies | Discovery-  Prevention and Control  Newer vector control strategies/vaccine, Vaccine development | Description-  Sero-epidemiology | |
| Hepatitis A | Development-Diagnostics  POCT for water testing, biosensor | Delivery-Antiviral trials  Vaccination policy | Delivery- Implementation/Operational Research  Availability of safe drinking water. Intersectoral coordination with Jal Shakti dept | Delivery-  Sero-surveillance  Performance of vaccine-vaccinated and non-vaccinated | |
| Measles | Description-Molecular epidemiology Complication, vulnerability | Description -Disease course and progression Studies  Vaccination Age, Seroprevalence of Maternal Ab, High risk pop identification and Intervention | Delivery-Prevention in targeted pop. (nomads, migratory groups, slums), community awareness | Delivery- Surveillance  Efficacy of vaccine, Diff b/w vaccine & natural immunity | |
| Rotavirus | Description-Vaccine efficacy, prevalence in mobile population, genotyping | Description-Disease course and progression Studies  Morbidity & Mortality at risk Population | Description-Investigation  Sero-prevalence, Geno surveillance, Vaccination coverage | Delivery-Vaccine coverage & efficacy | |

## **Supplement 6-** Overall comment and Suggestions for improvement

| Sr No | Overall Comment | Scope of Improvement |
| --- | --- | --- |
| 1 | “Good” | “Viral Culture should start in willing and performance-oriented Labs” |
| 2 | “Excellent” | “Preparedness for emerging viral epidemics” |
| 3 | “Excellent Workshop” | “Wastewater surveillance for Enterovirus” |
| 4 | “Very Good. Need to look forward in materializing he future agenda”. | “A pre workshop short video conference meet with participants will help to clarify certain angles of scope and objectives of the workshop, that will help improving decision making process.” |
| 5 | “It was very good experience” | “Research methodology.” |
| 6 | “The workshop was great as we have developed collaborative strengths and adequate interaction for future collaborative partnerships.” | “1. By prior sharing of workshop schedule and list of speakers, panel. also, we can include an assessment form during the workshop if applicable. 2. The collaborative project development, based on priority lists of viruses and research challenges.” |

## **Supplement 7-** Workshop Evaluation and Feedback

Structured feedback was sought form the participants regarding the workshop. Eighty (43%) participants provided feedback to the team (offline and online) on a scale of 0 to 5. Additional details are provided in supplement no.6.

The overall satisfaction with the workshop content was high, with a mean score of 4.6/5 corresponding to 92% of the maximum score. Participants also rated the relevance of the content (94%) and presenters’ effectiveness (94%) favourably. Satisfaction with workshop organization was also high, with mean score of 4.9/5 (97%). Some of the suggestions in the free text included preparedness for virus outbreaks capacity building, specific sero-surveillance for viruses like enterovirus in the East zone, a separate workshop for research methodology, and collaborative project development for identified viral diseases in the zone. Also, in the areas of improvement some useful suggestion included use of pre-recorded video message as a part of pre-workshop preparation for better understanding of the workshop objectives and proposed outcomes and research capability enhancement through contact sessions by ICMR-NIV.

Participant feedback on zonal workshops (n=80)

| **Zone** | **Total participants** | **Feedback received (%)** |
| --- | --- | --- |
| Central | 48 | 16 (33%) |
| East | 48 | 30 (63%) |
| North | 42 | 8 (19%) |
| South | 48 | 26 (54%) |
|  | 186 | 80 (43%) |
| **Feedback of the participants** | | |
| **Sr No** | **Questions-** | **Overall Score (Average)** |
| **1** | **Overall Satisfaction-**How satisfied are you with the content covered during the workshop? | 4.6 (92%) |
| **2** | **Content Relevance -** Please rate the relevance of the topics covered. | 4.7 (94%) |
| **3** | **Presenter Effectiveness -** How would you rate the effectiveness of the workshop presenters? | 4.7 (94%) |
| **4** | **Workshop Organization** - Rate the organization of the workshop sessions and activities | 4.9 (97%) |
| **5** | **Workshop Organization** - Were the workshop agenda and timing appropriate? | 5.0 (100%) |

## **Supplement 8-**List of experts and their affiliations

| **Sr. No** | **Zone** | **Domain** | **Area** | **Designation** | **Gender** | **Affiliation** | **Govt/ Non-Gov.** | **State** |
| --- | --- | --- | --- | --- | --- | --- | --- | --- |
| 1 | Central | Public Health | Health | Scientist G | M | ICMR-NIRTH, Jabalpur | Govt. | MP |
| 2 | Central | Virology | Non-Health | Scientist G | M | DRDE, Gwalior | Govt. | MP |
| 3 | Central | Virology | Health | Prof. and HOD | F | Microbiology, KGMU, Lucknow | Govt. | UP |
| 4 | Central | Clinical | Health | Addl. Prof. of Paediatrics | M | AIIMS, Bhubaneswar | Govt. | OD |
| 5 | Central | Veterinarian | Non-Health | Former JD, Principal Scientist | M | NIHSAD, Bhopal | Non-Govt. | MP |
| 6 | Central | Veterinarian | Non-Health | Principal Scientist | M | NIHSAD, Bhopal | Govt. | MP |
| 7 | Central | Microbiology | Health | Prof. and HOD | F | ICMR- BMHRC, Bhopal | Govt. | MP |
| 8 | Central | Environment Health | Health | Director | M | ICMR-NIREH, Bhopal | Govt. | MP |
| 9 | Central | Public Health | Health | Joint Director | M | NCDC, GoI, Bhopal | Govt. | MP |
| 10 | Central | Public Health | Health | WHO Representative | M | WHO, Bhopal | Non-Govt. | MP |
| 11 | Central | Environment Health | Health | Scientist G | M | ICMR-NIREH, Bhopal | Govt. | MP |
| 12 | East | Public Health | Health | Superintendent | M | Guwahati Medical College, Guwahati | Govt. | AS |
| 13 | East | Clinical | Health | Dean-Indian College of Physicians and President Elect | M | Association of Physicians of India | Non-Govt | WB |
| 14 | East | Clinical | Health | Head, Department of Infectious Diseases and Advanced Microbiology, | M | School of Tropical Medicine, Kolkota | Govt. | WB |
| 15 | East | Virology | Health | Head, Microbiology & State Level VRDL | F | Gauhati Medical College, Guwahati | Govt. | AS |
| 16 | East | Veterinarian | Non-Health | Joint Director | M | ICAR-IVRI Eastern Regional Station, Kolkata | Govt. | WB |
| 17 | East | Veterinarian | Non-Health | Associate Professor, Department of Microbiology | M | Assam Agricultural University, Johrat | Govt. | AS |
| 18 | East | Environment Health | Non-Health | Professor, Department of Zoology | F | Dibrugarh University | Govt. | AS |
| 19 | East | Epidemiology | Health | Executive Director | M | AIIMS Guwahati | Govt. | AS |
| 20 | North | Virology | Non-Health | Dean (Res.), Prof. & Head, Dept. Of Virology | F | PGIMER, Chandigarh. | Govt. | MP |
| 21 | North | Veterinarian | Non-Health | SIC | M | ICAR-IVRI, Mukteshwar, Uttarakhand | Govt. | UK |
| 22 | North | Clinical | Health | Director Principal and Professor | M | AIMS, Mohali, PB. | Govt. | PB |
| 23 | North | Public Health | Health | Professor and Head, Center for Community Medicine, | M | AIIMS, New Delhi | Govt. | Delhi |
| 24 | North | Public Health | Health | Consultant | M | Public Health & Climate change, Srinagar | Non-Govt. | J & K |
| 25 | North | Public Health | Health | Economist | M | CCU, Meerut, UP | Govt. | UP |
| 26 | North | Public Health | Health | Regional Director, | F | NCDC | Govt. | J & K |
| 27 | North | Microbiology | Health | Deputy Director, | F | NCDC, Delhi | Govt. | Delhi |
| 28 | North | Public Health | Health | SMO, WHO | M | Jammu, J & K | Govt. | J & K |
| 29 | North | Public Health | Health | Deputy Director | F | IDSP, Haryana | Govt. | HR |
| 30 | South | Microbiology | Health | Professor, Dept of Microbiology and Cell Biology, | M | IISc Bangaluru | Govt | KA |
| 31 | South | Clinical | Health | Prof & HOD, | F | Virology, NIMHANS | Govt | KA |
| 32 | South | Veterinarian | Health | Prof Veterinary College, | M | Hebbal, Bangaluru, Veterinary college | Govt | KA |
| 33 | South | Clinical | Health | Professor & Head of the Dept. Infectious Diseases | F | MAHE, Manipal | Pvt | KA |
| 34 | South | Epidemiology | Health | Professor & Head | M | Achutha Menon Centre for Health Science Studies,(An institution of national importance under DST, Govt. of India), Trivandrum, Kerala, India, PIN 695011. | Govt | KL |
| 35 | South | Veterinarian | Health | Scientist G | M | ICMR-Vector Control Research Centre | Govt | TN |
| 36 | South | Public Health | Health | Sr. Regional Director | M | Regional Office for Health & Family Welfare | Govt | KA |
| 37 | South | Environment Health | Non-Health | Scientist | F | Indian Institute for Human Settlements (IIHS), Bengaluru, Karnataka | NGO | KA |
| 38 | South | Public Health | Health | Regional Team Leader | M | WHO, Bangaluru | WHO | KA |
| 39 | South | Environment Health | Non-Health | Additional prof. Dept of Psycho-social support in Disaster Management | M | NIMHANS,Bangaluru | Govt | KA |

## **Supplement 9-** Post workshop activities

Following the workshop there are activities scheduled in year three of the PMABHIM-PHSPP for DLI 8. These activities will focus on the implications, for research and practice.

*1. Brainstorming Sessions***:**

To identify potential areas for multi-sectoral collaboration aimed at effectively preventing and controlling these prioritized diseases and to carry out research on the prioritized areas, meetings would be conducted with willing collaborators after a formal MoU with them.

**Proposed post-workshop activities-**

- Develop & implement action plan.
- MoU with willing collaborators
- Coordination, communication prevention & control (Outbreak Plans)
- Lab Training & Capacity building
- Joint Outbreak investigation
- Collaborative research on Prioritized disease and thematic areas- Joint Research Topic/Proposal development

*2. Understanding Stakeholder Roles:*

Roles and responsibilities of all represented stakeholders/collaborators for the prevention and control of emerging and re-emerging viral diseases will be made clear.

*3. Creating Roadmap for Preparedness:*

A roadmap for preparedness and response strategies for prioritized diseases will be developed including laboratory capacities for diagnostic testing, surveillance and research.

*4. Outcome Report:* A comprehensive report highlighting the workshop outcomes is planned to be produced to advocate for multi-sectoral coordination priorities.
